# An “over-fused middle cerebral artery” anomaly: a case report

**DOI:** 10.1186/s12883-021-02147-2

**Published:** 2021-03-17

**Authors:** Huifang Wang, Hui Liu

**Affiliations:** 1 Department of Neurology, Peking University Shougang Hospital, jinyuanzhuang No.9, Shijingshan, Beijing, China; 2 Department of Radiology, Peking University Shougang Hospital, Jinyuanzhuang No.9, Shijingshan, Beijing, China

**Keywords:** Middle cerebral artery, Anomaly, Over-fused MCA, Case report

## Abstract

**Background:**

MCA has several anomalies, such as accessory MCA, duplicated MCA and twig-like MCA, up to now all these reported anomalies were hypothesized to due to the failure in fusion of the primitive arterial network. No anomaly of over fused MCA has been reported.

**Case presentation:**

A 59- year- old male was hospitalized with a history of paroxysmal slurred speech and left side headache for a week, his blood pressure was 160/80 mmHg and he manifested mild incomplete motor aphasia at the time of admission. The head and neck CTA and DSA all presented a huge and tortuous left MCA, we diagnosed it an anomaly and termed it over-fused MCA. The patient’s speech impairment and headache were relieved by controlling his blood pressure.

**Conclusions:**

Such an anomaly of over-fused MCA is reported for the first time, it’s not needed to put special intervention on the anomaly of the patient temporarily, but more observation are needed.

## Background

There are several middle cerebral artery (MCA) anomalies such as accessory MCA, duplicated MCA, duplicated origin of MCA, fenestrated MCA, and aplastic or twig-like MCA (Ap/T-MCA), all these anomalies of MCA may form due to the failure in fusion of the primitive arterial network [1]. Here we report one case with unique anomaly of MCA which we named over-fused MCA. To the best of our knowledge this type of over-fused embryonic anomaly of the MCA has not been reported in the literature so far.

## Case presentation

A 59- year- old male was hospitalized on Sept. 4, 2017 with a history of paroxysmal slurred speech and left side headache for a week, with no limb numbness and weakness, no dizziness, diplopia and tinnitus, no conscious disorder, limb convulsions and urinary incontinence. The patient had no remarkable past history, personal history and family history. At the time of admission, blood pressure 160/80 mmHg, he manifested mild incomplete motor aphasia and no other neurological deficits.

**Fig. 1:**
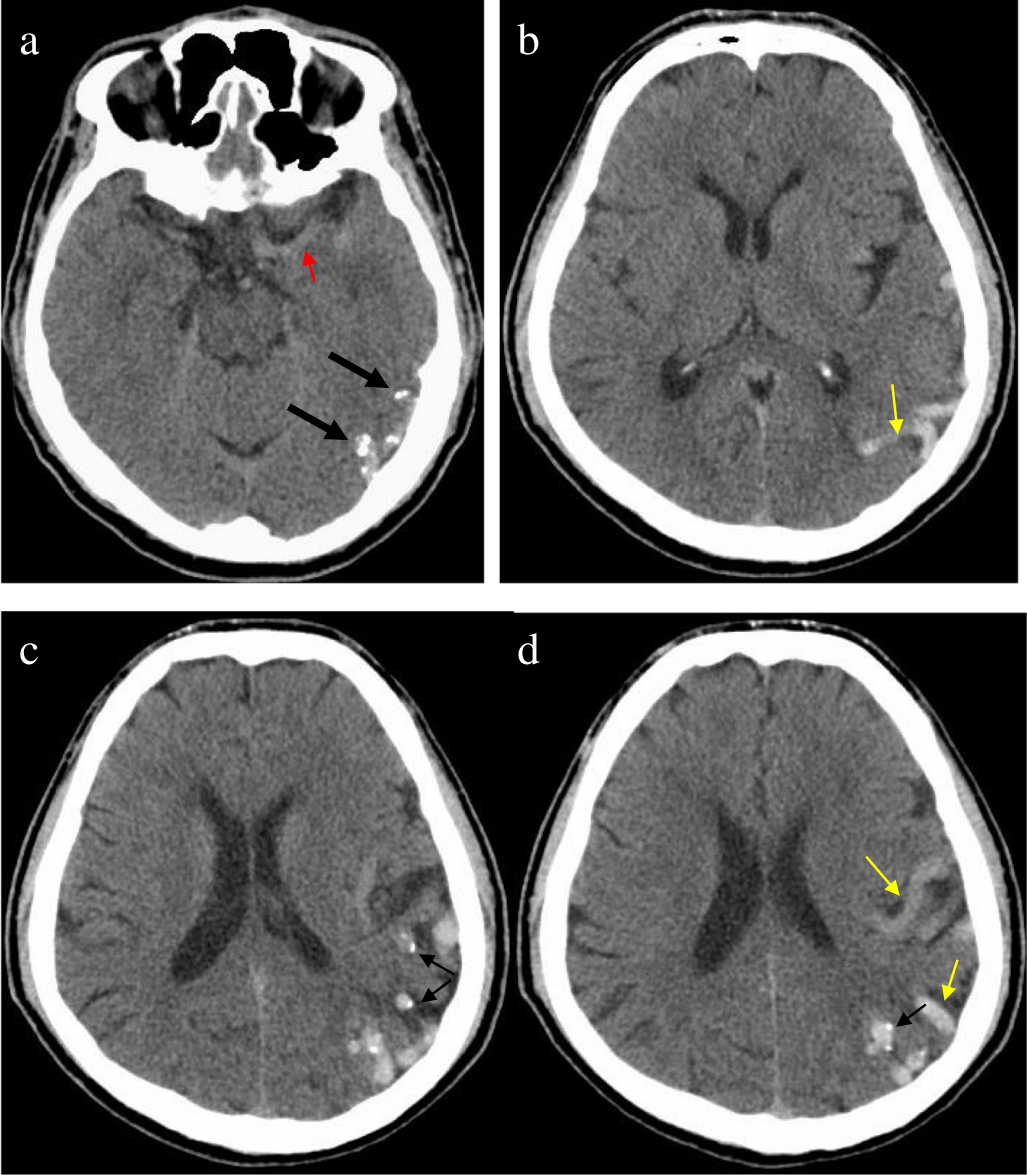
Head CT scan on admission showed presence of abnormal tortuous and dilated vessel in the left temporal and parietal lobe (yellow arrow), multiple punctuate calcification (thin black arrow) and nodular calcification (thick black arrow) and the tortuous and enlarged left MCA (red arrow)

**Fig. 2:**
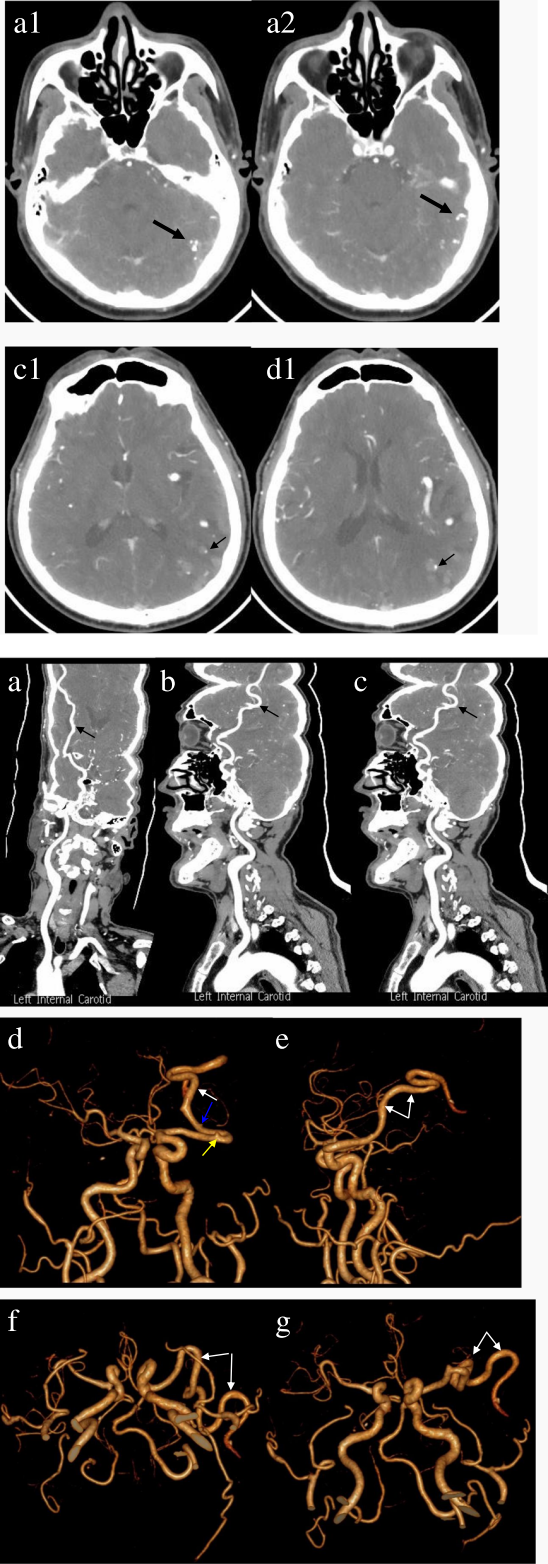
CT angiography (CTA) axial slices (**a1**, **a2**, **c1** and **d1**) also showed calcification at the same positions of the head CT scan: **a1** and **a2** to **a** (thick black arrow), **c1** to **c** and **d1** to **d** (thin black arrow), and the vessels are not enhanced. CTA with curved planar reformation (**a** ~ **c**) and with 3D reconstruction (**d** ~ **g**) showing that the left MCA is huge and tortuous and almost the same diameter from the beginning to the end (black arrow in **a** ~ **c** and white arrow in **d** ~ **g**). One small branch (yellow arrow) from the M1 segment and another small one (blue arrow) from the M2 segment

**Fig. 3:**
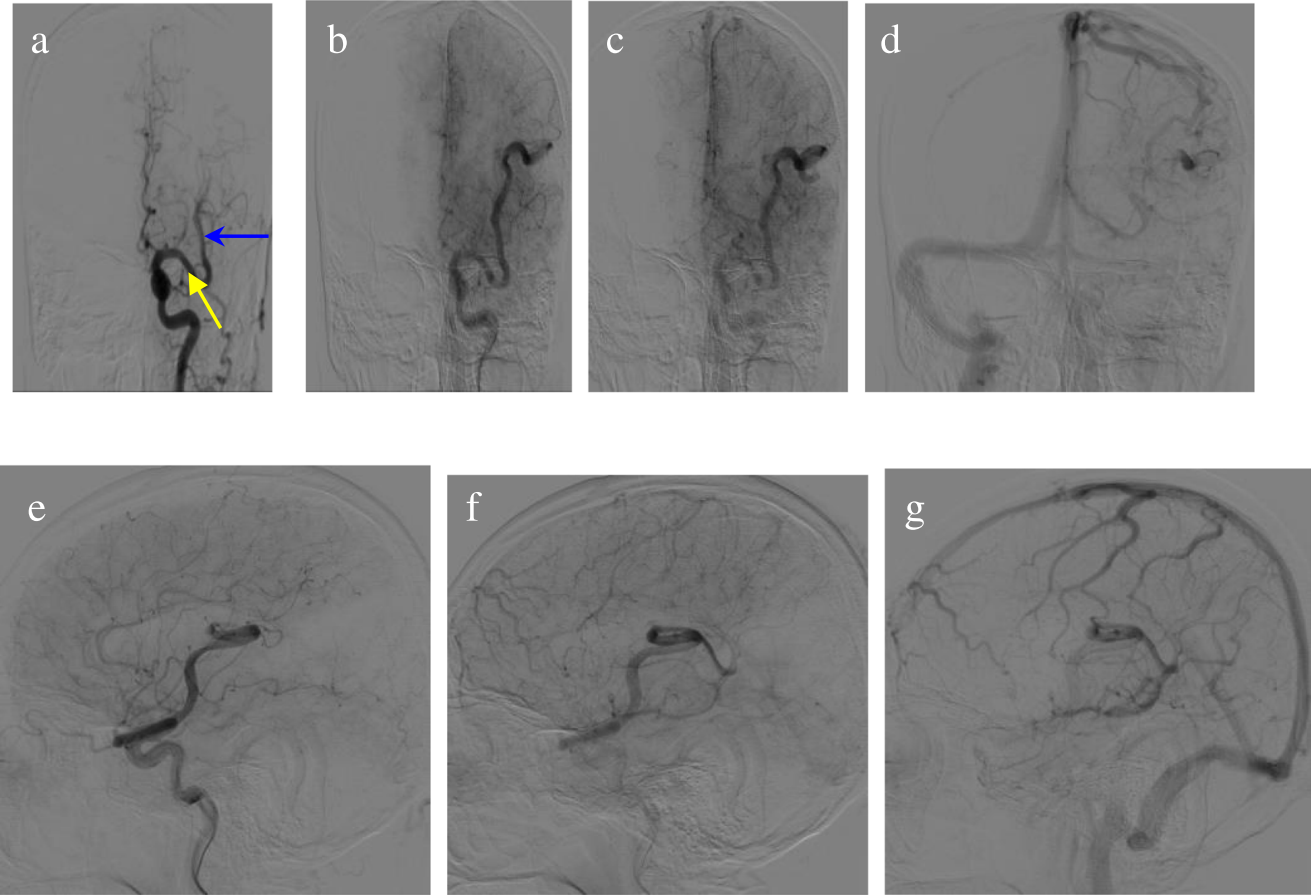
DSA. Left CCA injection. Lateral views (**e** ~ **g**) and AP views (**a** ~ **d**) showing the left MCA enlarged and tortuous, no vein in the late arterial phase and capillary phase; the huge MCA can be seen in the capillary phase (**b** and **e**), the vein phase (**c** and **f**) and the sinus phase (**d** and **g**); multiple lateral striate arteries at the proximal segment of M1, a small vessel (yellow arrow) rising at the distal segment of M1 and another small branch (blue arrow) rising at the proximal M2 segment

Outpatient head computed tomography (CT) scan prompted “left cerebral vascular malformation”. From the head CT scan, tortuous and dilated vessel could be seen in the left temporal and parietal lobe, multiple punctate and nodular calcification could be seen, and the left MCA was significantly tortuous and enlarged. The morphology of ventricles and cisterns were normal. No widening or deformation was observed in the lateral fissures (Fig. 1). Examination during hospitalization: Blood tests showed no abnormalities in lipids, blood glucose and homocysteine. There were also no significant abnormalities in electrocardiogram, echocardiography, abdominal B-ultrasound and electroencephalogram. Head Magnetic resonance imaging (MRI) presented that the distal branch of the left MCA was enlarged, there were tortuous blood vessels in the sulcus of the left temporal and parietal lobe with no other abnormality such as cerebral infarction or atrophy. Head and neck CT angiography (CTA) showed the distal branch of the left MCA enlarged, blood vessels and spotty calcification in the sulcus of temporal and parietal lobe, no obvious branches from the left MCA but one small from the M1 segment and another small one from the M2 segment, other vessels walking naturally with smooth and continuous walls and no stenosis. (Fig. 2) The patient’s digital subtraction angiography (DSA) showed that the left MCA was enlarged and tortuous, with nearly uniform diameter in the first 3/4 length and smaller diameter (probable 4/5 diameter of the first 3/4 segment) in the last 1/4 length. The filling flow was normal, the outflow was slow. The total MCA could be seen in the capillary phase, the whole vessel but the M1 segment could be seen in the venous phase, and about half of it could be seen in the sinus phase. No vein can be seen in the arterial and capillary phase. Multiple lateral striate arteries were rising at the proximal part of M1 segment, a small vessel rising at the distal part and a small branch rising at the M2 segment. (Fig. 3).

At first the patient was treated as ischemic stroke, after the investigation finished, no special treatment was given on the patient any more but controlling his blood pressure to no higher than 130/80 mmHg.The patient’s speech impairment was relieved shortly after hospitalization, and the same to his left headache. The left headache developed again about 1 year later and was relieved quickly by lowering blood pressure. In the late 2 years, there was no left headache, no speech impairment or other symptoms.

## Discussion and conclusion

MCA develops from multiple plexiform arterial twigs by fusion and regression [2]. The anomaly of MCA isn’t so common, accessory MCA, duplicated MCA, duplicated origin of MCA and fenestration of MCA have been reported and all of them were proposed due to the failure of fusion of the embryonic plexiform arteries [3–7].From 2015 on, cases of unfused or aplastic or twig-like MCA were reported [8, 9], the authors speculated that this kind of anomaly might be the persistent fetal arterial network of primitive MCAs. Briefly, all these anomalies of MCA occur at the proximal segment of MCA and were hypothesized due to the failure in fusion of the primitive arteries.

Our case has a totally normal right MCA, his left MCA has a huge main trunk from the beginning to the end with lateral striate arteries and two rather small branches supplying for the cortex. It is not subdivided into small arteries and capillaries like other large ones. The DSA showed that the feeding blood flow of the trunk is normal, the drainage /outflow is slow, there’s no vein in the arterial phase and capillary phase, and there are no compensatory collaterals supplying for the area of the left MCA. Normally [2] in a 12–14 mm embryo, just distal to the anterior choroidal artery, multiple plexiform arterial twigs rise and develop into lateral striate arteries and the trunk of MCA by fusion and regression, If the fusion fails, the anomalies of MCA mentioned above may be formed, we hypothesized that the multiple plexiform arterial twigs fused excessively, the huge MCA formed as in our case, so we gave it the term over-fused MCA.

Intracranial arteriovenous shunts (AVSs) mainly include arteriovenous malformations (AVMs), arteriovenous fistulas (AVFs) and developmental venous anomalies (DVAs), the anomaly in our case needs to be differentially diagnosed with AVSs, especially with AVMs and AVFs. AVMs are traditionally regarded as congenital lesions, primordial vascular channels fail to differentiate into mature intervening capillaries and veins, and instead create arteriovenous shunts without intervening capillaries [10, 11].AVM anatomy is essentially composed of one or more feeding arteries, a nidus, and one or more draining veins. The nidus connects to numerous vascular channels and is a high-flow system where arterial blood shunting occurs [12–14].By definition, there are no capillaries, abnormal short circuiting channels instead, between the feeding artery and the draining vein of AVF. Where AVF differ from AVM is that AVF lacks a true nidus. The pathophysiological mechanism of AVF is still unclear. It can result from trauma, previous surgery, or may be congenital [15]. In brief, the characters of AVM and AVF are arteriovenous shunt, high-flow arterial blood shunting and abnormal drainage, their critical character is that vein appears in arterial phase in DSA. It can be seen from the patient’s DSA that there is no vein or abnormal vascular mass in the artery and capillary stage, that’s to say there is no arteriovenous shunt. DSA also showed that the left MCA inflow and outflow are relatively slow, which may be because of the MCA’s large lumen and large blood volume and normal vein drainage, demonstrating no high-flow arterial blood shunting. There is an over fused MCA trunk in the patient, but there is no arteriovenous short circuit, no high-flow arterial blood shunting or abnormal vein. Therefore, arteriovenous malformation or arteriovenous fistula is not considered, and only a variation of artery is considered. Nevertheless, the thrombotic AVM should be in consideration in this case because that the calcified vessels in the posterior side of the temporal and parietal lobe in head CT (Fig. 1) are not enhanced in CTA (Fig. 2 A1 ~ D1). There are three points that do not support this diagnosis: 1. The patient’s DSA examination shows no thromboses in the vessels and also can exclude AVM, naturally can exclude thrombotic AVM; 2. The calcifications are located in the vascular wall rather than in the vascular cavity (images C1 and D1 in Fig. 2 showing more clearly); 3. The DSA shows that the blood flow of the huge left MCA is slow (illustrating by image A, B and C in Fig. 3), and the blood flow with enhancement agent has not reached the distal vessels during CTA scanning, which may be the reason why the vessels are not enhanced.

No CT perfusion or MR perfusion was carried out on the patient, but on the one hand the patient’s MRI didn’t show any cerebral atrophy which may imply no long period low-perfusion and on the other hand the CTA and DSA showed only two distal branches from the huge MCA which intimates over-perfusion nearly impossible, so we conjecture that the anomaly in this case has not been affecting the ipsilateral blood supply. To supporting this, of course, we’d propose a brain perfusion for the patient when there is another event on him. When blood pressure increased, local headache may be caused by vasodilation and traction, and also may caused by irritation of some factors such as serotonin, and paroxysmal reversible focal neurological disorder, such as speech impairment, may occur due to the local occupying effect. The drainage vessel is normal, but because the instantaneous blood perfusion exceeds the outflow, blood stasis appears at the end of the vessel, which may cause damage to the local vessel wall, such as calcification of the vessel wall. In one word, this anomaly has not brought serious and permanent damage to the patient, it is clear that there is no need to give special treatment to this patient at present. By controlling blood pressure, we can reduce the blood volume, decrease the tension to the vessel wall and the blood stasis at the terminal vessel, so as to minimize symptoms and other bad consequences. However, we still need to follow up the patient in order to know more about this MCA anomaly.

In conclusion, we present a case with unique anomaly of MCA, the anomaly which we termed over-fused MCA may form because of the over-fusion of the embryonic arterial network. No serious and permanent symptoms and signs, no special treatment is required to the anomaly at present, but more observation to this case are needed for it’s followed only for about 3 years.

## Data Availability

The datasets used and/or analysed during the current study available from the corresponding author on reasonable request.

## Abbreviations

MCA: Middle cerebral artery
Ap/T-MCA: Aplastic or twig-like MCA
CT: Computed tomography
MRI: Magnetic resonance imaging
CTA: CT angiography
AP: Anteroposterior
DSA: Digital subtraction angiography
CCA: Common carotid artery
AVS: Arteriovenous shunt
AVM: Arteriovenous malformation
AVF: Arteriovenous fistula
DVA: Developmental venous anomaly
